# Marine Natural Products as Anticancer Agents 2.0

**DOI:** 10.3390/md21040247

**Published:** 2023-04-17

**Authors:** Celso Alves, Marc Diederich

**Affiliations:** 1 MARE—Marine and Environmental Sciences Centre/ARNET-Aquatic Research Network, ESTM, Polytechnic of Leiria, 2520-630 Peniche, Portugal; 2 Department of Pharmacy, College of Pharmacy, Seoul National University, 1 Gwanak-ro, Gwanak-gu, Seoul 08826, Republic of Korea

Global cancer incidence and death are expected to increase to 28.4 million cases by 2040, despite efforts to understand cancer biology better and to improve its diagnosis and therapy [1]. Inappropriate therapeutic regimens and tumor recurrence due to the development of drug resistance are two major clinical challenges impacting poor patient outcomes [2]. Over the last few decades, the marine environment has become an important source of molecules with potent anticancer properties, exhibiting unusual chemical features and mechanisms of action. Twelve of seventeen drugs of marine origin approved by regulatory entities are used against cancer, and thirty-four of forty compounds in the marine pharmaceuticals pipeline indicate “cancer therapy” [3].

The Special Issue “Marine Natural Products as Anticancer Agents 2.0” (https://www.mdpi.com/journal/marinedrugs/special_issues/AnticancerAgents2, accessed on 11 April 2023) gathered eleven publications, including two reviews and nine research articles, about the anticancer activities of marine natural and/or derived products on in vitro 2D and 3D cellular models, and/or in vivo models of colorectal, breast, lung, myeloma, liver, leukemia, and melanoma cancers. Marine natural and/or derived products addressed herein were obtained from sponges, seaweeds, fungi, and soft corals. Those compounds exhibit great chemical diversity, such as terpenes, alkaloids, fatty acids, phenolics, polysaccharides, and peptides. Some compounds are halogenated. It is also relevant to highlight the key importance of “nature power creativity”, evidenced in this Special Issue as a supplier of scaffolds for the synthesis and molecular modeling of new anticancer drugs. Forty-seven analogs were synthesized based on the original structures of natural products of marine origin. These compounds exhibited high specificity and great affinity to interact with biomarkers related to specific intracellular signaling pathways involved in carcinogenesis, including cell cycle, oxidative stress, mitochondrial dysfunction, apoptosis, necrosis, stemness, angiogenesis, migration, and invasion.

Li and co-workers [4] provided a critical review of the application of fucoidans as cancer immunotherapy agents, discussing their action on different immune cells and as co-adjuvants of immunotherapeutic medicines. The prospects and challenges, as well as the current human clinical studies and available registered clinical trials using this type of compound against cancer, were also reported and discussed.

Vasarri and co-workers [5] compiled a critical overview focused on the last five years concerning the ability of marine natural products to prevent cancer cell migration and invasion, events related to the high mortality of patients with solid tumors. Forty-two compounds, including polysaccharides, peptides and proteins, polyphenols, and alkaloids, were identified as possessing migrastatic properties, limiting the pro-metastatic behavior of cancer cells in in vitro and in vivo models. This review provides a valuable guide for the pharmaceutical and scientific community as a starting point to develop new antimetastatic drugs based on the reported marine natural products.

The research and development of new drugs to reinforce the available therapeutic options will be crucial to improve the effectiveness of current cancer therapeutic regimens. Thus, the efforts to discover new chemical entities from natural origins that can inspire the development of new anticancer drugs, including synthesis and/or semi-synthesis approaches, are of the utmost relevance.

Sala and collaborators [6] isolated two novel free porphyrins, isabellin A and B, and the known compounds corallistin D and deuteroporphyrin IX from the marine sponge *Isabella* sp., previously collected in the hard substrate off Zuytdorp, Western Australia. The new free porphyrin, isabellin A, exhibited a marked cytotoxic effect on the NS-1 malignant myeloma cell line, similar to the positive control sparsomycin. Mohamed and co-authors [7] isolated five oxygenated cembrenoids, sarcoconvolutum A–E, from the soft coral *Sarcophyton* sp. collected in the Egyptian Red Sea. The cembrene diterpene sarcoconvolutum E exhibited the highest cytotoxicity on A549 (IC_50_: 49.70 µg/mL) and HSC-2 (IC_50_: 53.17 µg/mL) malignant cells derived from lung adenocarcinoma and squamous cell carcinoma of the oral cavity, respectively.

On the other hand, Patel and co-workers [8] explored the chemical structure of clavatadines, previously isolated from the marine sponge *Suberea clavate*, as a scaffold for developing new anticancer drugs. The authors synthesized a library of thirty-two pirocyclic clavatadine analogs, displaying a CC_50_ range between 0.4 and 12.3 µM on A-375 melanoma cells. The highest selectivity was exhibited by analogs 29 and 37, which activate caspases 3/7, key biomarkers of apoptosis. Lu and collaborators [9] also designed and semi-synthesized a series of fifteen derivatives of brefeldin A, a macrolactone biosynthesized by the marine fungus *Penicillium* sp. (HS-N-29), previously isolated from the medicinal mangrove *Acanthus ilicifolius*. Analog 7 (brefeldin A 7-*O*-2-chloro-4,5-difluorobenzoate) exhibited the smallest IC_50_ value (0.84 µM) on the human chronic myelogenous leukemia K562 cancer cell line. The mechanism of action underlying its cytotoxicity seems to be related to the blockage of the cell cycle, induction of apoptosis, inhibition of BCR-ABL phosphorylation, and downregulation of the expression of signaling molecules of the AKT pathway, including mTOR and p70S6K. Suksamai and collaborators [10] synthesized 5-*O*-(*N*-Boc-l-alanine)-renieramycin T (OBA-RT) from renieramycin T, an alkaloid commonly found in sponges and nudibranchs. OBA-RT can suppress cancer stem cell (CSC) activity and induce apoptosis in malignant lung cells, inhibiting Ak, which plays a key role in regulating CSC maintenance.

Alves and colleagues [11] studied the cytotoxic properties of three bromoditerpenes isolated from the red seaweed *Sphaerococcus coronopifolius*, named 12*R*-hydroxy-bromosphaerol, 12*S*-hydroxy-bromosphaerol, and bromosphaerol. Bromo groups, in their structure, characterize these terpenes due to bromine substitution, a rare chemical feature among secondary metabolites. The compounds displayed increased hydrogen peroxide production and induced apoptosis of MCF-7 cells derived from breast adenocarcinoma.

The anticancer activities of peptides (1–12), extracted and purified from the brown seaweed *Laminaria japonica* (LJPs), were evaluated through in vitro and in vivo models of liver cancer [12]. The LJP-1 peptide exhibited the highest anticancer activity, decreasing tumor growth in in vivo models. According to the evidence reported by Wu and co-workers [12], its mechanism of action seems to be related to the induction of cell death by caspase-dependent apoptosis, partly by inhibiting PI3K and MAPK signaling pathways and regulating the expression of cell cycle checkpoint proteins.

The development of resistance to anticancer drugs seriously threatens cancer treatment success. Distinct strategies have been designed to overcome these challenges, including drug synergism. Su and collaborators [13] explored the potential of a novel resolvin, 7*S*,15*R*-dihydroxy-16*S*,17*S*-epoxy-docosapentaenoic acid (diHEP-DPA), synthesized by cyanobacterial lipoxygenase from docosa–hexaenoic acid (DHA) to overcome the chemoresistance to 5-fluorouracil (5-FU) of colorectal cancer cells (CRCs) using in vitro and in vivo models. The new resolvin suppressed key events related to chemoresistance to 5-FU resulting from prolonged exposure, such as the enrichment of CSCs, the infiltration of tumor-associated macrophages, and the epithelial–mesenchymal transition in colorectal tumors. This evidence suggests that the therapeutic application of diHEP-DPA in combination with 5-FU-based chemotherapeutics against colorectal cancer is potentially effective.

Angiogenesis is the biological process responsible for forming new blood vessels, which play a key role in tumor growth, maintenance, and metastasis, being a valid target for tumor therapeutics. Dong and collaborators [14] evaluated the anti-angiogenesis activity of bis(2,3,6-tribromo-4,5-dihydroxybenzyl) ether (BTDE), a bromophenol previously isolated from the red seaweed *Rhodomela confervoides*. BTDE inhibited migration, invasion, tube formation, and the activity of matrix metalloproteinase 9 of human umbilical vein endothelial cells. It also prevented the migration, invasion, and vasculogenic mimicry formation of A549 lung adenocarcinoma cells. In vivo, BTDE blocked intersegmental vessel formation in zebrafish embryos.

Altogether, the eleven scientific papers published in this Special Issue provide an exciting overview of marine natural products as potential anticancer agents.

As Guest Editors, we acknowledge the efforts provided by all researchers and reviewers that participated in this Special Issue, the editorial board, and the *Marine Drugs* editorial office for their support and assistance. Special thanks go to Dr. Grace Qu.
